# The doctorate in clinical laboratory sciences: current status and outcomes

**DOI:** 10.1016/j.acpath.2025.100229

**Published:** 2025-12-01

**Authors:** Jose H. Salazar, Amber Asghar, Juan U. Rojo

**Affiliations:** Department of Clinical Laboratory Sciences, The University of Texas Medical Branch, Galveston, TX, USA

**Keywords:** Diagnostic error, Diagnostic management team, Doctorate in clinical laboratory sciences, Program outcomes

## Abstract

The Doctor of Clinical Laboratory Sciences (DCLS) degree was implemented at the University of Texas Medical Branch in 2016 with the goal of training laboratory professionals who can improve patient outcomes by participating as part of the healthcare team, providing input regarding the use of laboratory testing in clinical decision-making, and optimizing the utilization of laboratory tests. The University of Texas Medical Branch (UTMB)-DCLS program presented data regarding the implementation of the program from 2016 to 2021. This follow-up study presents the DCLS program outcomes to date. A DCLS alumni, clinical supervisor, and employer surveys were distributed to evaluate the effectiveness of the program and explore the role and patient-care impact of DCLS graduates. The majority of UTMB DCLS alumni secured employment before graduation or shortly thereafter and currently work in academic medical centers and reference laboratories as medical or laboratory directors. Clinicians rate the perceived contribution of DCLS graduates to clinical practice to be highly beneficial, and employers indicate a significant impact of DCLS graduates on patient outcomes. The results presented in this study demonstrate the active participation of DCLS in the healthcare team, thus supporting the need to invest in academic DCLS programs to further the growth of this advanced practice degree.

## Introduction

Despite the crucial role that laboratory testing plays in medical decision-making, there was a lack of professionals who could effectively bridge the gap between clinical laboratories and healthcare providers. This disconnect often contributes to improper laboratory test ordering and diagnostic errors, which can delay patient diagnosis and have serious consequences for patient safety and care quality.^1^^,^^2^ In an attempt to fill this critical gap in the healthcare system, the Doctorate in Clinical Laboratory Sciences (DCLS) academic program was developed to train advanced practice medical laboratory scientists who can support physicians in laboratory test selection and interpretation.^3^, ^4^, ^5^

As such, the DCLS program was implemented at the University of Texas Medical Branch (UTMB) in 2016, with the key objectives of training laboratory professionals to become expert consultants in laboratory testing, enhancing communication and collaboration between laboratory scientists and clinical care teams, reducing diagnostic errors through improved interpretation and application of laboratory tests results, advance evidence-based practice in laboratory medicine, and preparing leaders capable of driving innovation in clinical laboratory services.^6^, ^7^, ^8^

The initial report on the DCLS program's curriculum description and implementation covering the period from 2016 to 2020 provided valuable insights into the program's structure, demographics, and early outcomes.^4^ Briefly, with a 50% admission rate and a 21.8% attrition rate, the program graduated 15 students by December 2020. Surveys of faculty physicians indicated that DCLS students contributed positively to clinical rounds, and a majority of students expressed interest in leading or participating in diagnostic management teams (DMTs) upon graduation. These early outcomes suggested that the DCLS program effectively trains doctoral-level advanced practice medical laboratory scientists. However, the long-term impact of the program and its sustainability remained uncertain, necessitating a follow-up study.

Now, five years after the initial report, we present a comprehensive follow-up study to evaluate the long-term effectiveness of the DCLS program. This study focuses on how the program has shaped graduate outcomes, enhanced their contributions to patient care, and demonstrated its potential for broader integration into the healthcare system. Specifically, we examine the program's progress and outcomes over the past five years, including DCLS graduate job placement and areas of practice, while also identifying opportunities for program improvement and expansion. Through this analysis, we aim to provide a deeper understanding of the DCLS program's role in advancing clinical laboratory sciences and its capacity to address the persistent challenge of diagnostic errors in healthcare.

## Materials and methods

A quality improvement/quality assurance survey to evaluate the long-term outcomes of the DCLS program at the University of Texas Medical Branch, Galveston, since its inception in 2016, was conducted. The academic program's admission, attrition, and graduation rates from 2021 to 2025 were obtained from program records and administrative databases.

The effectiveness of the program was evaluated by gathering student perspectives, graduate career outcomes, scholarly contributions, and employer feedback to evaluate the extent to which the DCLS program has contributed to advanced-level clinical laboratory science practice. Likewise, perspectives from clinicians involved in DCLS training and DCLS employers were evaluated.

A DCLS alumnus, a DCLS clinical faculty, and a DCLS employer anonymous survey were created using QuestionPro Survey Software. Each survey was distributed via direct email to alumni, clinicians, and employers who have been involved in the DCLS program since its inception in 2016 through December 2024. For the DCLS alumni survey, 46 DCLS students who have graduated from the UTMB DCLS program since 2019 were invited to participate. Alumni demographics, employment status, job duties, scholarly outcomes, and satisfaction with their DCLS degree were collected. For the DCLS clinical supervisor survey, 28 faculty physicians who were involved in the supervision of DCLS students during their clinical rotations were invited to participate via email. Data gathered from the clinical supervisors included their medical specialty, time overseeing DCLS students, likelihood of mentoring future DCLS students, and the assessed contribution of DCLS to clinical practice. For the DCLS employer survey, alumni voluntarily provided the contact information of their supervisors, which was used to distribute the survey via email. The DCLS employer survey gathered data regarding the duration DCLS alumni have been employed at their place, assessed the DCLS competencies in their practice, and indicated their likelihood of hiring future DCLS graduates. All surveys were made available for 90 days, after which the data was analyzed using QuestionPro analytics.

## Results

### DCLS program admissions, attrition, and graduation

Over the five-year follow-up period, the UTMB DCLS program showed steady growth. The overall acceptance rate for 2021 to 2025 was 32% (47 out of 147), with an overall attrition rate of 11%. A total of 46 students who enrolled between 2019 and 2024 successfully completed the program.

### DCLS alumni survey

**Fig. 1:**
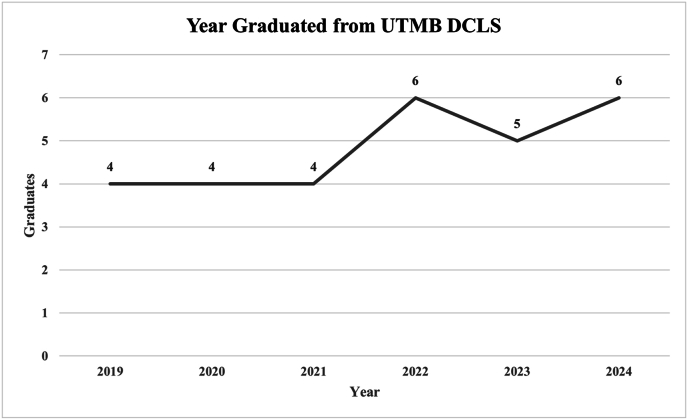
Survey respondents' year graduated from UTMB DCLS program. DCLS: Doctor of Clinical Laboratory Sciences; UTMB: University of Texas Medical Branch.

A total of 46 UTMB DCLS alumni were invited to participate, of whom 29 completed the survey. Balanced responses from alumni who graduated in a given year since 2019 were received, with most respondents having graduated in 2022 and 2024 (Fig. 1).

**Fig. 2:**
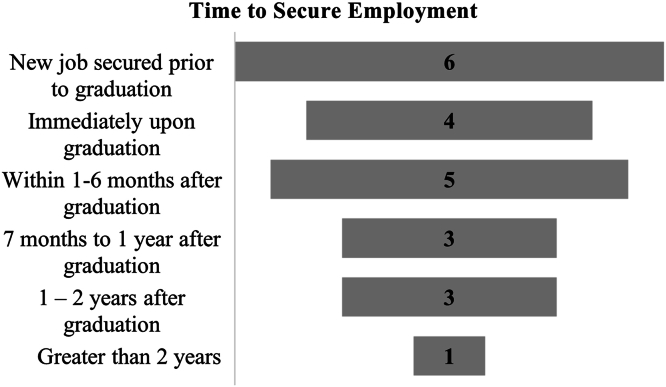
DCLS graduate time to secure employment. DCLS: Doctor of Clinical Laboratory Sciences.

Most DCLS alumni reside in the state of Texas. The majority of respondents were in the 35–44 age group (Table 1). For employment outcomes, six students secured a new job before graduation (Fig. 2) and 75.8% of respondents expressed that their DCLS degree contributed to obtaining a new job offer (Table 2).

**Table 1:** DCLS alumni demographic characteristics (N = 29).

Invited to participate	46
*Completed the survey*	29
Male	13 (44.8%)
Female	16 (55.2%)
*Age group*	
25–34	2 (6.9%)
35–44	21 (72.4%)
45–49	4 (13.8%)
Over 50	2 (6.9%)
*Race and ethnicity*	
White	16 (55.1%)
Asian	8 (27.6%)
Alaskan or American Indian	2 (6.9%)
Black or African American	2 (6.9%)
Declined to Answer	1 (3.5%)
Hispanic, Latino, or Spanish Origin	4 (13.8%)
Not Hispanic, Latino, or Spanish Origin	25 (86.2%)
*State of Residence*	
Texas	17 (58.6%)
New York	3 (10.3%)
Illinois	2 (6.9%)
Massachusetts	2 (6.9%)
Wisconsin	2 (6.9%)
Delaware	1 (3.4%)
Kansas	1 (3.4%)
Maryland	1 (3.4%)

DCLS: Doctor of Clinical Laboratory Sciences.

**Table 2:** DCLS alumni employment outcomes (N = 29).

	Accepted new job after DCLS degree	DCLS degree contributed to new job offer	Relocated as a result of earning DCLS	Earned additional degrees	Passed board certifications
Yes	21 (72.4%)	22 (75.8%)	6 (20.7%)	0	10 (34.5%)
No	8 (27.6%)	1 (3.5%)	23 (79.3%)	29 (100%)	19 (65.5%)
Not applicable	**-**	6 (20.7%)	**-**	**-**	**-**

DCLS: Doctor of Clinical Laboratory Sciences.

The DCLS practitioners are primarily employed in academic medical centers (37.5%), followed by reference laboratories (20.8%), and four-year universities (20.8%) (Table 3). Among these, the most common job titles held by DCLS professionals are Medical Laboratory Director (24.1%) and Assistant Professor (20.8%). Several respondents reported holding more than one of the surveyed job titles. (Table 4).

**Table 3:** DCLS professional place of work (N = 24).

Work environment	Number
Academic medical center	9 (37.5%)
Reference laboratory	5 (20.8%)
Four-year university	5 (20.8%)
Community college	3 (12.5%)
Community of general hospital	1 (4.2%)
Private laboratory	1 (4.2%)

DCLS: Doctor of Clinical Laboratory Sciences.

**Table 4:** Current job titles held by DCLS alumni (N = 29).

Current job title	Number (%)
Medical or laboratory director	7 (24.1%)
Assistant professor	5 (20.8%)
Clinical assistant professor	3 (10.3%)
Laboratory manager	3 (10.3%)
Associate professor	2 (6.9%)
Laboratory consultant	2 (6.9%)
Program director	2 (6.9%)
Assistant director	1 (3.4%)
Assistant vice president	1 (3.4%)
Associate laboratory director	1 (3.4%)
Clinical architect	1 (3.4%)
Clinical associate professor	1 (3.4%)
Director of transfusion services	1 (3.4%)

Note: Respondents could select multiple job titles. DCLS: Doctor of Clinical Laboratory Sciences.

Among all respondents (N = 29), ten DCLS alumni decided to pursue additional board certifications, with the National Registry of Certified Chemists (NRCC) Clinical Chemist being the top choice. Other board certifications including NRCC Toxicology Chemist and the American College of Histocompatibility & Immunogenetics (ACHI) (Table 5).

**Table 5:** Number of DCLS graduates who acquired board certifications (N = 29).

Type board of certification	Number (%)
NRCC clinical chemist	8 (27.6%)
NRCC toxicology chemist	1 (3.4%)
ACHI director	1 (3.4%)
No additional certification	19 (65.6%)

ACHI,: American College of Histocompatibility & Immunogenetics; DCLS: Doctor of Clinical Laboratory Sciences; NRCC: National Registry of Certified Chemists.

As part of their job responsibilities, DCLS practitioners reported a variety of duties, including providing training, monitoring laboratory test utilization, managing patient care, evaluating healthcare delivery, recommending and interpreting laboratory tests, and preparing materials for clinicians (Table 6).

**Table 6:** Job responsibilities held by DCLS professionals (N = 29).

Duties performed as DCLS professional	Number
Teach and mentor students/residents in a college or university setting	18 (10.4%)
Monitor laboratory test utilization	16 (9.2%)
Overall laboratory management	15 (8.7%)
Implement and evaluate healthcare delivery quality measures supported by quality theory and research	14 (8.1%)
Manage patient care by recommending or interpreting laboratory tests	13 (7.5%)
Prepare and present instructional materials for clinicians	13 (7.5%)
Perform and supervise procedures that require highly developed technical skills	12 (7%)
Plan, organize, direct, and supervise research projects	12 (7%)
Utilize health informatics and technological systems to provide laboratory consults	12 (7%)
Write and publish manuscripts in peer-reviewed journals	12 (7%)
Supervision of laboratory personnel	11 (6.4%)
Conduct research in collaboration with others, write papers, and submit grants	8 (4.6%)
Develop an independent line of research and apply for extramural funding	3 (1.7%)
Prepare and present instructional materials for patients	2 (1.2%)
K-12 education	0

Note: Respondents could select more than one job responsibility. DCLS,: Doctor of Clinical Laboratory Sciences.

**Fig. 3:**
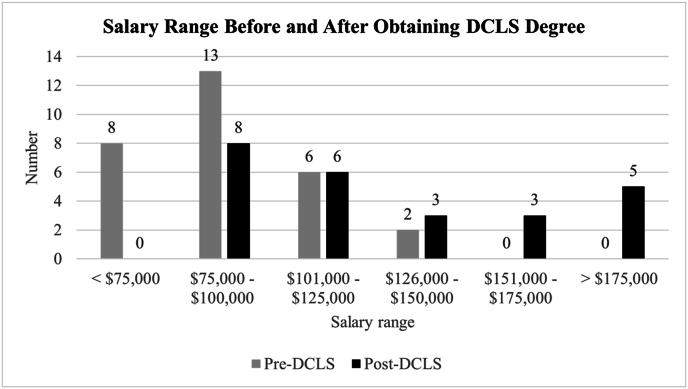
Annual salary range of DCLS professionals before and after obtaining their DCLS degree. *Note:* Only 25 respondents reported their post-DCLS salary. DCLS: Doctor of Clinical Laboratory Sciences.

Participants reported higher salaries after obtaining their DCLS degree, with a shift into higher salary ranges. Before earning the DCLS, 21 respondents reported annual salaries below $100,000. After obtaining the DCLS degree, 8 respondents reported salaries between $75,000 and $100,000, with 17 respondents reporting salaries greater than $100,000 (Fig. 3).

As part of their research training, DCLS students are required to complete a thesis as an outcome of scholarly activity. The DCLS alumni research work resulted in publications in a peer-reviewed journals (26.5%) or was presented as an abstract or poster presentation (17.7%) (Table 7). Overall, the majority of DCLS alumni reported being very satisfied (65.5%) or satisfied (31%) with their degree (Table 8).

**Table 7:** Outcome of DCLS research work (N = 29).

DCLS thesis outcome	Number
Published in a peer-reviewed journal	9 (26.5%)
Presented as an abstract or poster	6 (17.7%)
Submitted for publication and currently under review	3 (3.8%)
Presented as an oral presentation at a state or national conference	1 (3%)
Currently preparing for submission to a journal	7 (20.6%)
Not applicable	8 (23.5%)

Note: Respondents could select more than one outcome. DCLS: Doctor of Clinical Laboratory Sciences.

**Table 8:** DCLS alumni degree of satisfaction with their DCLS degree (N = 29).

Satisfaction with DCLS degree	Number
Very satisfied	19 (65.5%)
Satisfied	9 (31%)
Not satisfied	1 (3.5%)
Very unsatisfied	0

DCLS: Doctor of Clinical Laboratory Sciences.

### DCLS clinical faculty survey

A total of 17 clinicians involved in the direct supervision of DCLS students during their clinical rotations completed the survey. The majority of respondents supervised DCLS students in Internal Medicine, followed by Clinical Pathology, with most clinicians having more than 1 year of experience supervising DCLS students. Out of the 17 physicians surveyed, 16 expressed their willingness to mentor DCLS students again (Table 9).

**Table 9:** DCLS clinical supervisor survey report (N = 17).

Completed the survey	17
*Specialty*	
Internal medicine	9 (52.9%)
Clinical pathology	6 (35.3%)
Infectious diseases	1 (5.9%)
Surgical critical care	1 (5.9%)
*Time Overseeing a DCLS*	
<6 months	2 (11.8%)
6 months to 1 year	1 (5.9%)
1–3 years	6 (35.3%)
3–5 years	4 (23.5%)
>5 years	4 (23.5%)
*Willing to Mentor/Supervise DCLS Again*	
Yes	16 (94.1%)
No	1 (5.9%)

DCLS,: Doctor of Clinical Laboratory Sciences.

The perceived contribution of DCLS students to clinical practice as reported by clinicians, indicated that most agreed or strongly agreed that DCLS enhancing their access to laboratory resources, provides appropriate and effective consultation, increasing their understanding of clinical laboratory tests, and that the DCLS student was beneficial to their clinical practice (Table 10).

**Table 10:** Clinician perceived contribution of DCLS students to clinical practice (N = 17).

	Enhanced access to laboratory resources	Provided appropriate and effective consultation	Increased my understanding of clinical laboratory tests	Beneficial to my clinical performance
Strongly agree	8	8	5	7
Agree	7	6	6	8
Neutral	2	3	5	2
Disagree	0	0	1	0
Strongly disagree	0	0	0	0

DCLS: Doctor of Clinical Laboratory Sciences.

### DCLS employer survey

Five employers of DCLS practitioners participated in the survey. Three employers had supervised the work of DCLS graduates for 1–3 years, and two for more than 3 years. Employers rated DCLS professionals to have Very Good or Excellent competency for all assessed tasks, including providing clinical expertise, problem-solving, knowledge of laboratory management, interpreting patient information, and managing laboratory test ordering and interpretation (Table 11). Employers of DCLS professionals reported that DCLS graduates have a significant impact on patient outcomes, and all employers expressed being Likely (N = 1) or Very Likely (N = 4) to hire future DCLS graduates.

**Table 11:** DCLS employer perceived contribution of DCLS professionals (N = 5).

Statement	4	5	N/A
Clinical expertise and application of laboratory sciences	1	4	0
Problem-solving and critical thinking in clinical decision-making	3	2	0
Knowledge of laboratory management and operations	1	4	0
Gathering and interpreting patient information	2	1	2
Managing patient care through laboratory test ordering and interpretation	1	2	2
Preparing and presenting instructional material for clinicians	1	3	1
Implementing quality measures based on research and quality theory	1	3	1
Utilizing health informatics and technological systems for laboratory consultations	0	2	3
Building empathetic and cooperative relationships with patients and healthcare workers	0	3	2
Applying ethical principles in the clinical laboratory sciences	0	5	0

DCLS: Doctor of Clinical Laboratory Sciences.

## Discussion

In this follow-up study, we described the outcomes of the UTMB DCLS program over the last five years. The information presented in this report provides valuable information regarding alumni satisfaction with their DCLS degree and offers perspectives from clinicians and employers regarding their interactions with DCLS professionals. The information presented herein also serves to support other institutions that want to implement similar programs to educate medical laboratory scientists at the doctoral level.

The UTMB DCLS program graduates an approximately equal number of male and female students. As admission to the DCLS program requires applicants to have a minimum of three years of work experience in the clinical laboratory, the majority of DCLS alumni are in the 35 to 44-year age group.

As our DCLS program is based in Texas, it was expected that most of the DCLS graduates would reside in the state of Texas. It was observed that the majority of DCLS graduates secured new employment no later than 6 months after graduation. This finding suggests that DCLS graduates are in demand, often securing employment before graduation or soon thereafter.

The primary goal of the DCLS program is to prepare doctorate-level professionals who bridge communication between the clinical laboratory and physicians. In our UTMB training program, this goal is supported through competency-based clinical training and DMTs. Upon graduation, the reported job responsibilities of DCLS professionals show that they actively contribute to improving diagnostic practices by monitoring laboratory test utilization and supporting patient care through test recommendations and interpretation. The results from the DCLS alumni survey further indicate that graduates serve as leaders in diagnostic medicine, with roles in laboratory management, supervision, and education. This indicates that the program's objectives are being achieved, supported by published work highlighting the value of a dedicated professional in reducing unnecessary testing and ultimately improving patient outcomes.^9^^,^^10^ Moreover, earning a DCLS degree was associated with improved salary outcomes as graduates reported higher annual salary ranges after completing the program.

Although the DCLS curriculum is not designed to confer specific postgraduation certifications, the observed interest of graduates in pursuing NRCC board certification, a Centers for Medicare & Medicaid Services (CMS)-recognized board, suggests that many aspire to serve as high-complexity laboratory directors (HCLDs). Under CMS regulations (42 CFR §493.1443), HCLDs must hold a doctoral degree in a chemical, physical, biological, or clinical laboratory science and be certified by a board approved by the U.S. Department of Health and Human Services. At the UTMB, both prospective and graduating DCLS students have expressed their interest in CMS-recognized pathways that would allow them to work as HCLDs. These professional aspirations are often discussed during admissions interviews and continue throughout the program in advising and mentorship sessions. We thus have observed a strong interest among graduates in pursuing nationally recognized certifications to complement their DCLS degree, either to qualify as a clinical consultant or to serve as an HCLD.

These latter findings highlight the importance of DCLS programs providing resources for board exam preparation and collaborating with accrediting boards to ensure graduates have a clear pathway toward CMS-recognized credentials. A larger multi-institutional study is warranted to further assess how programs can align training and resources with CMS requirements for board certification and laboratory leadership roles. Such a study can also assess the extent to which DCLS graduates establish DMTs or similar strategies at their institutions.

As part of their DCLS training, students are required to complete a thesis-based research project. The students’ research findings have been disseminated in peer-reviewed journals and presentations. This outcome is significant for the DCLS professionals working in a four-year university setting (20.8%) and community college (12.5%) as dissemination of research is an expected outcome for promotion and academic growth. Overall, DCLS graduates are satisfied with the impact of their degree on career placement and job duties.

Clinicians reported a high level of satisfaction with the impact of the DCLS on clinical practice. The majority of physicians reported more than one year of experience collaborating with DCLS professionals. Accordingly, most clinicians indicated that the DCLS role enhances access to laboratory resources and provides effective consultation, contributing to an improved understanding of clinical laboratory tests. The single case of a clinician not willing to mentor or supervise a DCLS student in the future can be attributed to an isolated negative experience, likely influenced by individual factors such as personality differences.

Likewise, employers value the contribution of DCLS to their sites of employment. According to their employers, DCLS are rated as excellent in providing clinical expertise and applying laboratory science, and they possess the skills to enhance laboratory decision-making, optimize test utilization, and interpret patient information. As such, all employers were likely to hire future DCLS graduates as they acknowledge DCLS graduates' significant impact and contribution to patient outcomes. Because employer contact information was voluntarily provided by DCLS alumni, we only received responses from five employers. Thus, the limited information collected may not be representative of all employers. Additionally, as this is a single-institution study, results may not be fully generalizable to other DCLS programs. Larger multi-institutional surveys are needed to more comprehensively evaluate the impact of DCLS practice.

Overall, DCLS graduates pursue diverse opportunities in various settings, including hospital laboratories, reference laboratories, and institutions of higher education. Their advanced training prepares them to participate in consulting and leadership roles as well as to assess research design. As the healthcare sector is experiencing a significant shortage of medical laboratory professionals and physicians, advanced practice providers, such as the DCLS, can help meet the need for enhancing patient care by integrating laboratory science knowledge with clinical decision-making, with the ultimate goal of optimizing patient care through better use and understanding of laboratory diagnostic tests.^11^, ^12^, ^13^

## Conclusions

With the CMS ruling recognizing the DCLS as qualified to serve as an HCLD, DCLS graduates actively participate in improving diagnostic practices by providing evidence-based patient care as part of the healthcare team, bringing clinical practice and laboratory services closer together.^14^ The DCLS graduates act as consultants and leaders in bringing innovation to laboratory medicine. As more academic institutions integrate the DCLS program as part of their degree offerings, the number of DCLS graduates will naturally increase. Universities that offer DCLS degrees, as well as professional organizations, should continue to advocate in support of the DCLS profession and its impact on healthcare practice.^15^ The DCLS degree presents a promising opportunity for individuals seeking to advance in the field and meet the evolving demands of healthcare. As such, healthcare systems are encouraged to continue integrating DCLS professionals by including the DCLS degree as one of the degree requirements for job postings in laboratory consultation services, management, and directorship.^16^ Likewise, insurance companies should implement a proper venue for billing DCLS interpretative and consultative work. The results presented in this follow-up study demonstrate a promising outlook for DCLS professionals.

## Funding

This research received no specific grant from any funding agency in the public, commercial, or not-for-profit sectors.

## Declaration of competing interest

The authors declared no potential conflict of interest regarding the research, authorship, and/or publication of this article.

## References

[1] Laposata M. (2018). Obtaining a correct diagnosis rapidly in the United States is associated with many barriers not present in other countries. Am J Clin Pathol.

[2] Laposata M. (2004). Patient-specific narrative interpretations of complex clinical laboratory evaluations: who is competent to provide them?. Clin Chem.

[3] Nadder T.S. (2013). The development of the doctorate in clinical laboratory science in the U.S. EJIFCC.

[4] Salazar J.H., Zahner C.J., Freeman V.S., Laposata M. (2021). The doctorate in clinical laboratory sciences: a new curriculum to enhance the connection of the laboratory to health care providers. Acad Pathol.

[5] Greiner A.C., Knebel E., Institute of Medicine (US) Committee on the Health Professions Education Summit (2003). Health Professions Education: A Bridge to Quality.

[6] Leibach E.K. (2008). The doctorate in clinical laboratory science: a view of clinical practice development. Clin Lab Sci J Am Soc Med Technol.

[7] Laposata M., Cohen M.B. (2016). It's our turn: implications for pathology from the institute of medicine's report on diagnostic error. Arch Pathol Lab Med.

[8] Verna R., Velazquez A.B., Laposata M. (2019). Reducing diagnostic errors worldwide through diagnostic management teams. Ann Lab Med.

[9] Sarkar M.K., Botz C.M., Laposata M. (2017). An assessment of overutilization and underutilization of laboratory tests by expert physicians in the evaluation of patients for bleeding and thrombotic disorders in clinical context and in real time. Diagn Berl Ger.

[10] Neilson E.G., Johnson K.B., Rosenbloom S.T. (2004). The impact of peer management on test-ordering behavior. Ann Intern Med.

[11] Aghaei M., Khademi R., Bahreiny S.S., Saki N. (2024). The need to establish and recognize the field of clinical laboratory science (CLS) as an essential field in advancing clinical goals. Health Sci Rep.

[12] Rahim F. (2024). The effect of unintended shortage in technical resources on the quality of endpoint clinical laboratory diagnosis. Clin Chem Lab Med.

[13] Maganty A., Byrnes M.E., Hamm M. (2023). Barriers to rural health care from the provider perspective. Rural Remote Health.

[14] Clinical Laboratory Improvement Amendments of 1988 (CLIA) Fees. Histocompatibility, personnel, and alternative sanctions for certificate of waiver laboratories. Fed Regist. 2023. December 28;88(248):89976–90044. https://www.federalregister.gov/documents/2023/12/28/2023-28170/clinical-laboratory-improvement-amendments-of-1988-clia-fees-histocompatibility-personnel-and. [Accessed 6 April 2025].

[15] Montoya I.D., Kimball O.M. (2007). A marketing clinical doctorate programs. J Allied Health.

[16] Beck S., Doig K. (2007). Are new CLS practitioners prepared to stay?. Clin Lab Sci J Am Soc Med Technol..

